# Vegetable and fruit juice enhances antioxidant capacity and regulates antioxidant gene expression in rat liver, brain and colon

**DOI:** 10.1590/1678-4685-GMB-2016-0159

**Published:** 2017-03-20

**Authors:** Linhong Yuan, Jinmeng Liu, Jie Zhen, Yao Xu, Shuying Chen, Nicholas Van Halm-Lutterodt, Rong Xiao

**Affiliations:** 1 School of Public Health, Capital Medical University, Beijing, P.R. China

**Keywords:** antioxidant, biomarkers, fruit and vegetable, gene expression

## Abstract

To explore the effect of fruit and vegetable (FV) juice on biomarkers of oxidative damage and antioxidant gene expression in rats, 36 adult male Wistar rats were randomly divided into control, low FV juice dosage or high FV juice dosage treatment groups. The rats were given freshly extracted FV juice or the same volume of saline water daily for five weeks. After intervention, serum and tissues specimens were collected for biomarker and gene expression measurement. FV juice intervention increased total antioxidant capacity, glutathione, vitamin C, β-carotene, total polyphenols, flavonoids levels andglutathione peroxidaseenzyme activity in rat serum or tissues (p < 0.05). FV juice intervention caused reduction of malondialdehyde levels in rat liver (p < 0.05) and significantly modulated transcript levels of glutamate cysteine ligase catalytic subunit (GCLC) and NAD(P)H:quinone oxidoreductase l (NQO1)in rat liver and brain (p < 0.05). The results underline the potential of FV juice to improve the antioxidant capacity and to prevent the oxidative damage in liver, brain and colon.

## Introduction

Epidemiological studies indicate that the consumption of fruits and vegetables (FV) reduces the risk of various diseases related to oxidative damage, such as cancer, cardiovascular diseases, diabetes and Alzheimer's disease (Stanner *et al.*, 2004; Collins, 2005; Lau *et al.*, 2007; Montonen *et al.*, 2007). Dietary intake of antioxidants obtained from natural sources, such as fruit and vegetables, is considered to be relatively safe without presenting undesirable side effects (Tchantchou *et al.*, 2004).

The beneficial effects of FV consumption are largely attributed to the antioxidants contained in FV, such as vitamins, carotenoids, and phenolic phytochemicals (Liu, 2003, 2013a,b; 2013;). Furthermore, compared with antioxidant vitamins, such as vitamin E, FV-derived flavonoids are proved to be more efficient in antagonizing oxidative damage *in vivo* and *in vitro* (Rao, 2003). In addition to the direct scavenging of free radicals, antioxidants in FV were able to protect against reactive oxygen species (ROS)-mediated oxidative damage by elevating cellular antioxidant capacity (Rubió, *et al.*, 2013). A series of dietary intervention studies carried out in humans and in animals also showed that a FV-rich diet can cause the elevation of peroxide-detoxifying enzymes’ activities including superoxide dismutase (SOD), glutathione peroxidase (GSH-Px) and catalase (CAT) (Dragsted *et al.*, 2004; Kasdallah-Grissa *et al.*, 2007; Polidori *et al.*, 2009).

Phase II antioxidant enzyme gene expression and activity were proved to be inducible by FV-derived antioxidants through the trans-activation of the Nuclear Factor Erythroid-Derived 2-like 2 (NFE2L2 or Nrf2) signaling pathway (Owuor*et al.*, 2002). Itoh *et al.* (2010) first described that the activation of Nrf2 initiated the transcription of phase II antioxidant enzymes, and the expression of genes encoding antioxidant enzymes, such as glutathione S-transferase Mu 1 (GSTM1), glutathione S-transferase A2 (GSTA2), glutathione S-transferase P 1 (GSTP1), NAD(P)H:quinone oxidoreductase l (NQO1), glutamate cysteine ligase catalytic subunit (GCLC), glutamate cysteine ligase regulatory subunit (GCLM), was reported to be regulated by Nrf2 (Itoh *et al.*, 2010). Additionally, in experimental animals the expression of antioxidant genes regulated by the Nrf2 signaling pathway was reported to play important roles in antagonizing the pathogenesis of oxidative damage-related diseases (Aleksunes and Manautou, 2007).

Individuals who consumed an FV-rich diet appear to have a lower risk of many chronic diseases (de Morais *et al.*, 2013). However, the accurate mechanism by which an FV-rich diet decreases the risk of oxidative damage-related diseases is still unclear. Previous studies carried out in experimental animals mainly focus on the effects of FV (or their juices) on the levels of antioxidant biomarkers (Duthie *et al.*, 2006). However, few studies explored the profile of oxidative damage-related biomarkers and antioxidant gene expression in different organs in animals. Thus, the aim of the present study was to elucidate whether FV juice supplementation influences oxidative damage-related biomarkers in animal serum and tissues. We also investigated the influence of FV juice intervention on the antioxidant gene expression in rat liver, brain and colon. The current study will provide basic data to uncover the mechanisms of how a FV-rich diet leads to health-promoting effects *in vivo*.

## Materials and Methods

### Reagents

Total RNA Kit was obtained from Promega (Madison, WI, USA). DNA primers were purchased from NanHuaKeAoTeconolgoy Development Ltd. (Beijing, China), and stored as 100 pM/μL stock solution at −20 °C. AmpliTaq Gold TM DNA polymerase, reverse transcriptase kit was purchased from Promega. Total antioxidant capacity (T-AOC), malondialdehyde (MDA), glutathione (GSH), GSH-Px and total SOD enzyme activity measurement kits were purchased from Nanjing Jiancheng Company (Nanjing, China). Purified (100%) grape juice was obtained from HuiYuan Juice Ltd. (Beijing, China). All the vegetables used in the juice preparation were purchased from a local supermarket. All the reagents used for β-carotene and flavonoids measurement were purchased from Sigma Chemical Co. (Sigma, USA).

### Vegetable juice preparation

Six vegetables consumed frequently by Chinese residents (carrot, celery, tomato, purple onion, broccoli, and green pepper) were selected for the vegetable juice. Briefly, vegetables were chopped, and then the juice was extracted in a juice making machine (Hurom Slow Juicer, product No. HU-600WN, Gyeongsangnam-do South Korea). The resulting juices were used for the dietary intervention.

### Experimental animals

Thirty six adult male Wistar rats (SPF class, weighing 250-300 g) were provided by the Chinese Academy of Sciences. The rats were housed at room temperature with a 12 h light-dark cycle and were given free access to tap water. After a 1-week adaptation, the animals were randomly divided into three groups of 12 rats each: control group (saline water), and low FV juice dosage and high FV juice dosage intervention groups. We organized a diet menu (see Supplementary Material Table S1), in which two vegetable juices and grape juice were used daily for dietary intervention (The nutrients contained in fruit and vegetables are listed in Table S2). The menu was rotated every three days. The animals were fed with standard laboratory chow throughout the whole study. Body weight of the animals was measured weekly. The animal experiments were approved by the Ethic Committee on Experimental Animal of Capital Medical University (NO. 2013-x-9) and conducted following the guidelines established by the Chinese Committee on Experimental Animal Supervision.

The Chinese Nutrition Society (CNS) recommends 500 g of vegetables and 400 g of fruit per day for healthy adult Chinese residents (Sun, 2012). For a standard adult [body weight (bw) 60 kg], the average fruit and vegetable intake is 6.67 g/kg of bw and 8.33 g/kg of bw respectively. Therefore, in the present study, those amounts were used as the low dosage of FV juice dietary intervention to mimic the normal FV intake of humans. A dosage five-fold higher than of the low dosage (33.35 g/kg of bw for fruit and 42.75 g/kg of bw for vegetable) was used for the high dosage of FV juice dietary intervention. According to the body weight of each rat, we finally determined the administration volume of grape juice and each vegetable juice. The FV juice was administered by oral gavage for 5 weeks.

### Measurement of biomarkers

At the end of the experiment, the rats were food-deprived for 12 h, and a blood sample was collected from the arteria cruralis. Briefly, the rats were immobilized and anaesthetized. Skin in the inguen was cut and the arteria cruralis was exposed by blunt dissection. A syringe was used to draw blood. Blood was centrifuged at 1500 rpm for 5 min. Liver, brain and colon tissues were quickly excised, rinsed with 0.9% sodium chloride solution, frozen in liquid nitrogen and stored at −80 °C until further analysis. Liver, brain and colon homogenates were prepared at1:10 (w/v) in ice cold phosphate buffered saline (PBS, pH 7.4) and centrifuged at 3800 *g* at 4 °C for 10 min. The supernatant was used for biomarker measurement. T-AOC, MDA, GSH, vitamin C content, GSH-Px and total SOD enzyme activities in tissues and serum were measured by using kits from NanJingJianCheng Bioengineering Institute (Nanjing, China). The procedure was performed according to the manufacturer's instruction. Total polyphenol content in rat serum was detected according to the protocol of Slinkard and Singleton. (1997). Liquid chromatography-tandem mass spectrometry (LC-MS) was applied to measure the β-carotene and flavonoids content in rat serum following the protocol of Andreoli *et al.* (2004) and Li *et al.* (2004)

### Reverse transcription-polymerase chain reaction (RT-PCR)

Total RNA of liver, colon, and brain tissues was purified by using Trizol reagent (Invitrogen, Carlsbad, CA, USA), and reverse transcription was performed by a reverse transcriptase kit purchased from Promega. Briefly, double-stranded DNA was synthesized from 2 μg of total RNA, and the 2 μg cDNA obtained was used as a template for PCR. The mRNA levels of GSTP1, GSTA2, GSTM2, NQO1, GCLC, GCLM and β-actin (used as an invariant control) in liver, colon, and brain tissues were measured. The forward and reverse primer sequences were as follows: GSTP1, F: 5’ AGA TGT CTG GCT TCA AGG 3’; R: 5’ TTC ACC ATA TCC ACC AAG 3’; GSTA2, F: 5’ ACA GAC CAG AGC CAT TCT 3’; R: 5’ TTT GGT CCT GTC TTT TGC 3’; GSTM2, F: 5’ TTT GGT CCT GTC TTT TGC 3’; R: 5’ CAA AGT CAG GGC TGT AGC 3’; GCLM, F: 5’ ATG CCA CCA GAT TTG ACT G 3’; R: 5’ CAC TCC TGG GCT TCA AG 3’; GCLC, F: 5’ CCG AGT TCA ACA CAG TGG 3’; R: 5’ TCC TTC CTC TGG GTT GG 3’; NQO1, F: 5’ GAA GAAGAA AGG ATG GGA G 3’; R: 5’ GCC TTC CTT ATA CGC CAG A 3’; β-actin, F: 5’ AGA TCC TGA CCG AGC GTG GC 3’; R: 5’ CCA GGG AGG AAG AGG ATG CG 3’. After 35 cycles, amplification products were electrophoresed on a 2.0% agarose gel. Then, FluorChem FC2 software (Alpha Innotech, CA, USA) was used to capture the images and analyze the gray value of the respective mRNA levels in each group.

### Statistical analysis

Data are reported as means ± SE and analyzed with SPSS 13.0 software (SPSS Inc., Chicago, USA). Statistical analysis was performed using one-way analysis of variance (ANO**V**A); followed by a post hoc Duncan's multiple range test. A *P* value of less than 0.05 was considered statistically significant.

## Results

### Body weight and organ coefficient

The rats gained weight from week 0 to week 5. All animals were physically healthy, and body weight was not different among groups during the experiment (data not shown). The isolated brain, heart, spleen, livers, kidneys, and testes were weighed, and the organ coefficient in different treatment groups was compared after the dietary intervention for five weeks. However, no difference was detected among groups (Table S3).

### Serum biomarkers

As shown in Table 1, FV juice dietary intervention increased serum vitamin C, β-carotene, GSH, total polyphenols and flavonoids content significantly when compared with the control group (p < 0.05). Compared with the low FV juice dosage group, the high FV juice group showed significantly increased serum vitamin C and β-carotene (p < 0.05). Additionally, compared to the control group, dietary FV juice increased serum GSH-Px enzyme activity and T-AOC (p < 0.05) without affecting total serum SOD enzyme activity (p > 0.05). The high FV juice dosage caused a higher serum GSH-PX enzyme activity than low FV juice dosage (p < 0.05). As for flavonoids content in serum, FV juice significantly increased the content of luteolin, naringenin and kaempferol in serum (p < 0.05). Compared with the low FV juice dosage group, high FV juice dosage supplementation had a much stronger effect in enhancing serum luteolin and naringenin levels (p < 0.05).

**Table 1:** Effects of fruit and vegetable (FV) juice dietary intervention on serum antioxidant parameters.

Biomarkers	Control	Low dosage	High dosage
		FV juice	FV juice
MDA (nmol/mL)	9.38 ± 0.40	9.30 ± 0.16	9.15 ± 0.26
GSH (μmol/L)	57.47 ± 17.42	120.47 ± 37.52*	101.98 ± 30.95^**^
T-AOC (U/L)	9.09 ± 1.55	9.53 ± 1.38	10.73 ± 1.84*
GSH-Px (activity U/L)	435.66 ± 23.23	446.35 ± 25.44	475.97 ± 25.78* ^,**^
SOD (U/mL)	23.49 ± 4.51	24.30 ± 8.81	25.24 ± 7.36
Vitamin C (μg/mL)	8.58 ± 0.11	8.73 ± 0.08	9.41 ± 0.09* ^,**^
β-carotene (μg/mL)	1.97 ± 0.08	3.50 ± 0.17*	4.95 ± 0.32* ^,**^
Total polyphenol (mg GA/mL)	0.16 ± 0.01	0.19 ± 0.06*	0.18 ± 0.06
Flavonoids (ng/mL)			
Luteolin	38.70 ± 2.20	57.14 ± 5.14*	82.40 ± 8.89* ^,**^
quercetin	ND	40.49 ± 6.67	61.83 ± 10.79
Naringenin	821.60 ± 32.75	940.99 ± 24.38*	1105.9 ± 30.16* ^,**^
Apigenin	ND	ND	ND
Kaempferol	ND	12.49 ± 2.44	31.88 ± 4.98^**^
Hesperetin	5.50 ± 1.67	10.85 ± 2.26	15.07 ± 4.98

Data were reported as means ± SE, (n = 12 for each group).MDA: malondialdehyde;GSH: glutathione; T-AOC: total antioxidant capacity; GSH-Px: Glutathione peroxidase; SOD: Superoxide dismutase; ND: Not detected. ANOVA was applied for data analysis followed by LSD test and Dunnett T3 test.

* p < 0.05 compared with control group; *p < 0.05compared with low dosage FV juice dietary intervention group.

### Biomarkers in rat liver

FV juice intervention had no effects on GSH content and SOD enzyme activity in rat liver (p > 0.05) (Table 2). However, a significant decrease of MDA content in rat liver tissue was detected in low and high FV juice dietary intervention groups (p < 0.05). Compared with the control group, five weeks of FV juice dietary intervention increased T-AOC level (p < 0.05) and GSH-Px enzyme activity in rat liver tissue (p < 0.05).

**Table 2:** Effects of fruit and vegetable (FV) juice dietary intervention on MDA, GSH, T-AOC levels, GSH-Px and SOD enzyme activities in rat liver tissue.

Biomarkers	Control	Low dosage	High dosage
		FV juice	FV juice
MDA (nmol/mg liver protein)	0.56 ± 0.06	0.36 ± 0.05*	0.36 ± 0.02^**^
GSH (mg/g liver protein)	46.16 ± 4.52	47.99 ± 7.53	45.00 ± 4.73
T-AOC (U/mg liver protein)	0.68 ± 0.03	0.931 ± 0.02*	0.898 ± 0.09*
GSH-Px (activity U/mg liver protein)	31.78 ± 5.85	63.98 ± 8.21^**^	51.53 ± 6.79*
SOD (U/mg liver protein)	107.22 ± 13.31	125.23 ± 18.58	110.85 ± 12.30

MDA: malondialdehyde; GSH: glutathione; T-AOC: total antioxidant capacity; GSH-Px: Glutathione peroxidase; SOD: Superoxide Dismutase. Data were reported as means ± SE, (n = 12 for each group).

* p < 0.05,*p < 0.01 compared with control group.

### Biomarkers in rat brain

As shown in Table 3, FV juice intervention had no effect on T-AOC, MDA content, and SOD enzyme activity in rat brain tissue when compared with the control group. An elevation of GSH content and in GSH-Px enzyme activity was detected in rat brain tissues in the high FV juice dosage group compared with the control group(p < 0.05).

**Table 3:** Effects of fruit and vegetable (FV) juice dietary intervention on MDA, GSH, T-AOC levels, GSH-Px and SOD enzyme activities in rat brain tissue.

Biomarkers	Control	Low dosage	High dosage
		FV juice	FV juice
MDA (nmol/mg brain protein)	0.99 ± 0.25	0.91 ± 0.15	1.01 ± 0.19
GSH (mg GSH/g brain protein)	70.71 ± 10.22	75.49 ± 10.88	81.08 ± 10.55*
T-AOC (U/mg brain protein)	2.09 ± 0.43	1.84 ± 0.34	1.938 ± 0.36
GSH-Px (activity U/mg brain protein)	53.16 ± 12.82	61.98 ± 11.84	64.40 ± 8.69*
SOD (U/mg brain protein)	105.81 ± 16.80	92.80 ± 9.62	101.557 ± 14.25

MDA: malondialdehyde; GSH: glutathione; T-AOC: total antioxidant capacity; GSH-Px: Glutathione peroxidase; SOD: Superoxide dismutase. Data were expressed as mean ± SE, (n = 12 for each group).

* p < 0.05 compared with control group.

### Biomarkers in rat colon

As shown in Table 4, FV juice intervention had no effects on MDA content, GSH-Px and SOD enzyme activities in rat colon tissue compared with control group. Although we observed an increasing trend of T-AOC, no statistically significant difference was detected when compared with the control group. Moreover, an elevated GSH content was detected in the FV juice-treated group (p < 0.05).

**Table 4:** Effects of fruit and vegetable (FV) juice dietary intervention on MDA, GSH, T-AOC levels, GSH-Px and SOD enzyme activities in rat colon tissue.

Biomarkers	Control	Low dosage	High dosage
		FV juice	FV juice
MDA (nmol/mg colon protein)	0.43 ± 0.07	0.40 ± 0.10	0.45 ± 0.09
GSH (mg/g colon protein)	36.41 ± 10.38	42.61 ± 9.37	53.28 ± 10.26* ^,**^
T-AOC (U/mg colon protein)	7.16 ± 1.22	8.12 ± 1.31	8.21 ± 1.59
GSH-Px (activity U/mg colon protein)	120.48 ± 15.20	124.88 ± 13.34	123.94 ± 14.31
SOD (U/mg colon protein)	96.90 ± 18.73	97.35 ± 19.65	94.97 ± 19.17

MDA: malondialdehyde; GSH: glutathione; T-AOC: total antioxidant capacity; GSH-Px: Glutathione peroxidase; SOD: Superoxide dismutase. Data were expressed as mean ± SE, (n = 12 for each group).

* p < 0.01 compared with control group; *p < 0.05 compared with low dosage FVjuice group.

### Antioxidant gene expression in rat liver

**Figure 1:**
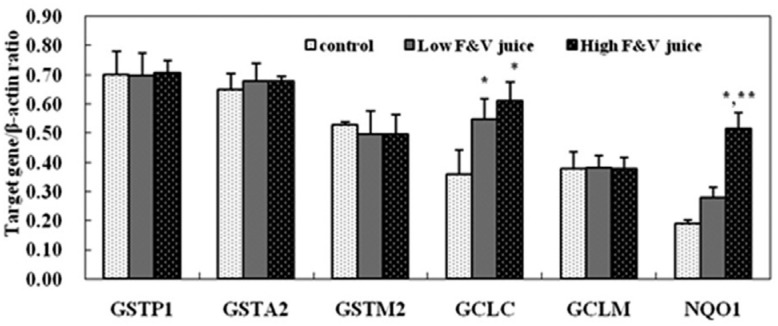
Expression of antioxidant genes in rat liver (n = 12). Rats received saline water (control), low fruit and vegetable (FV) juice dosage, and high FV juice dosage for 5 weeks. The animals were sacrificed and liver samples were collected for target gene expression detection by RT-PCR method. β-actin was used as endogenous reference gene, and every experiment was repeated for 3 times. Values represent relative band density (normalized to the endogenous reference gene β-actin). *p < 0.01 compared with control group; *P < .05 compared with low dosage FV juice group.

High FV juice dosage supplementation up-regulated the mRNA levels of GCLC and NQO1 compared with the control group (p < 0.01). However, five weeks of FV juice dietary intervention had no effects on the mRNA levels of GSTP1, GSTA2, GSTM2, and GCLM in rat liver tissue (Figure 1).

### Antioxidant gene expression in rat brain

**Figure 2:**
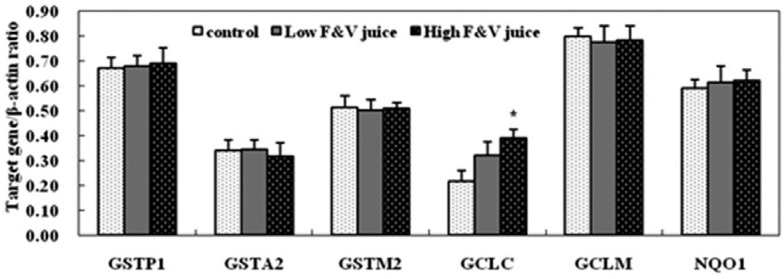
Expression of antioxidant genes in rat brain (n = 12). Rats received saline water (control), low fruit and vegetable (FV) juice dosage, and high FV juice dosage for 5 weeks. The animals were sacrificed and brain samples were collected for target gene expression detection by RT-PCR method. β-actin was used as endogenous reference gene, and every experiment was repeated for three times. Values represent relative band density (normalized to the endogenous reference gene β-actin). *p < 0.05 compared with control group.

The mRNA levels of GCLC and NQO1 in brain were up-regulated by FV juice intervention. Compared with the control group, high FV juice dosage intervention significantly increased GCLC gene expression of (p < 0.05). Although the mRNA levels of GSTM2, GCLM and GSTA2 exhibited a slightly decreasing trend in rat brain tissue, no statistically significant difference was detected among groups (Figure 2).

### Antioxidant gene expression in rat colon

**Figure 3:**
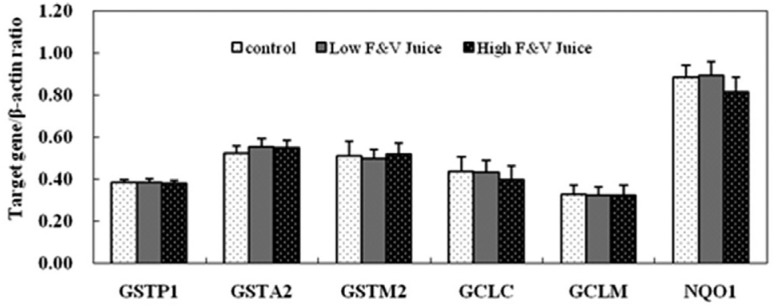
Expression of antioxidant genes in rat colon (n = 12). Rats received saline water (control), low fruit and vegetable (FV) juice dosage, and high FV juice dosage for 5 weeks. The animals were sacrificed and colon samples were collected for target gene expression detection by RT-PCR method. β-actin was used as endogenous reference gene, and every experiment was repeated for three times. Values represent relative band density (normalized to the endogenous reference gene β-actin).

The mRNA levels of GCLC and NQO1 in the high dosage FV juice-treated group appeared to be slightly down-regulated when compared with the control and low dosage FV juice groups. However, no statistically significant difference was detected among groups. The mRNA levels of GSTP1, GSTA2, GSTM2, and GCLM in colon tissue of rats that received FV juice intervention had a similar level to that in the control group (Figure 3), and no statistical significance was detected.

## Discussion

Our results indicate that five weeks of FV juice dietary intervention increased serum GSH, vitamin C, β-carotene, T-AOC levels and GSH-Px enzyme activity. Other than antioxidant vitamins and enzymes, polyphenols were also suggested to contribute to the antioxidative preventive effects of fruit and vegetable. In the current study, we found that five weeks of FV juice intervention increased total polyphenol concentration in the low dosage FV juice intervention group. Moreover, an elevated serum content of flavonoids, especially luteolin, naringenin and kaempferol, was observed after five weeks of FV juice dietary intervention. These results suggest that the increase of multiple antioxidants contributes to the enhancing effects of FV juice on antioxidant capacity.

Nonetheless, in the current study, no difference in serum MDA level and SOD enzyme activity was detected among groups. We further detected changes in oxidative damage-related biomarkers in rat liver, brain and colon tissues, and differences in these biomarkers were observed. In rat liver, five weeks of FV juice supplementation decreased the MDA content and increased T-AOC. However, we did not observe such changes in rat brain and colon tissues. Decreased lipid peroxidation was observed as, indicated by a significant decrease in MDA after FV juice treatment. These results indicate that the FV juice was efficient in protecting liver cells from oxidative damage. Liver is the primary vital organ involved in the detoxification of xenobiotics and is subjected to stress during the detoxification. ROS are well known and important risk factors for liver diseases (Muriel, 2009; Ha *et al.*, 2010). Since membrane phospholipids are a major target of oxidative damage. MDA is considered an indicator of free radical damage through membrane lipid peroxidation (Chang*et al.*, 2000; Chenand Bakhiet, 2006). Numerous studies indicate that fruit- and vegetable-derived flavonoids, such as apigenin, luteolin, quercetin, naringenin and kaempferol, protect low-density lipoprotein from oxidation and inhibit carbon tetrachloride (CCl_4_)-induced rat liver NADPH-dependent microsomal lipid peroxidation (Cholbi *et al.*, 1991; Añón *et al.*, 1992). In the current study, we detected an increase of several flavonoids in rat serum after FV juice intervention. This might explain the decline of MDA level in liver tissue. Additionally, it has been reported in many studies that carotenoids, such as β-carotene and lycopene, have antioxidant effects against lipid peroxidation in rat liver (Chen *et al.*, 1993; Martin*et al.*, 1996; Whittaker *et al.*, 1996). Therefore, liver might be a valuable target organ for carotenoids. In the present study, the carotenoid-rich vegetables carrot, tomato and broccoli were used for dietary intervention, which contributed to the significant increase in serum β-carotene concentration after FV juice intervention. We propose that in addition to antioxidative vitamins, carotenoids from FV juice might be associated with the tissue-specific changes of MDA levels in rat liver.

GSH is an important non-enzymatic antioxidant, which protects cells from oxidative damage. Hence, the levels of GSH reflect the antioxidant capacity and the severity of oxidative damage in the body. Inconsistent with the findings in rat brain and colon tissues, we found that FV juice intervention had no effect on GSH content in liver tissue. The possible explanation may be that, as the first line detoxification occurs in the liver, it consumes more antioxidants, including GSH, to reduce the ROS stress and avoid the influence of ROS on second line tissues such as brain and kidney. This explanation was further evidenced by the increased T-AOC and GSH-Px enzyme activity, and decreased MDA in liver tissue.

Elevated GSH-Px enzyme activity was detected in high FV juice dosage-fed rat liver and brain tissues but not in colon tissues. In contrast, SOD enzyme activity did not differ among groups in all tissues. We currently cannot explain the different profiles of GSH-Px and SOD enzyme activities found in rat tissues. However, according to published data, we speculate that SOD enzyme activity might be easily induced when the experimental animals are under pathological or stressful status (Galisteo*et al.*, 2004; Sugiura*et al.*, 2013).

Moreover, we found that rat liver tissue was more sensitive to FV juice treatment, which was evidenced by the significant decrease in MDA content, T-AOC and GSH-Px enzyme activity after low FV juice dosage intervention. However, in brain and colon tissues the changes in oxidative damage-related biomarkers were only detected in the high dosage FV juice treatment group. The possible explanation for this phenomenon might be that, as exogenous compounds, food-derived antioxidants will be first metabolized by the liver, the presence of enteric and enterohepatic recycling allows the antioxidants to be accumulated in liver overtime, resulting in the liver's priority in benefiting from FV juice intervention. These results indicate that the liver is probably the target organ for oxidative damage protection from an FV-rich diet.

An important finding of this study was that FV juice supplementation significantly affected NQO1 and GCLC mRNA expression in rat liver. At the same time, the mRNA levels of other antioxidant genes, including GSTP1, GSTA2, GSTM2, GCLM, remained similar to those of the control group. Accumulated data indicate that antioxidative phytochemicals from fruits and vegetables can activate the Keap1/Nrf2 system by multiple mechanisms and regulate the Nrf2 pathway downstream of antioxidant gene expression (Middleton Jr*et al.*, 2000; Qin and Hou., 2016). In the current study, we detected different regulation effects of FV juice treatment on antioxidant gene expression. These results were consistent with the data from Soyalan*et al.* (2011) who found that apple juice up-regulated GSH-Px and NQO1 gene expression in rat liver tissue without affecting SOD1, SOD2, GCLM gene expression. Together with our findings, these results indicate that the expression levels of some antioxidant genes, such as NQO1 and GCLC genes; GSH-Px and NQO1 genes, are highly correlated and present similar mechanisms of induction. The differences in gene expression might be ascribed to the fact that the transcription of ARE-driven genes is only in part regulated by Nrf2, and ARE motif may also interact with other DNA-binding proteins (Favreau and Pickett, 1995; Jaiswal, 2000).

Oxidative stress can dramatically alter neuronal function and ultimately cause neuron degenerative diseases. As a result, considerable research has been aimed at reducing the effects of oxidative stress in order to prevent Alzheimer's disease progress by using free radical scavengers (Chakrabarti *et al.*, 2013). Brain-accessible antioxidants may potentially provide a means for implementing this therapeutic strategy and delaying the disease onset. In the present study, we found that FV juice intervention significantly increased GCLC gene expression, and slightly increased NQO1 gene expression. GCLC, a rate-limiting enzyme in glutathione synthesis, is an isoenzyme of γ-glutamylcysteinesynthetase (Lu, 2013). GSH was regarded as a key antioxidant involved in the oxidative damage defense in brain tissue. A reduction in GSH level was also detected in patients diagnosed with mild cognitive impairment (MCI) or Alzheimer's disease (Jomova *et al.*, 2010; Mandal *et al.*, 2012). Together with our findings on the increased GSH content in brain tissue we speculate that the regulation of GCLC gene expression caused by FV juice intervention plays an important role in protecting brain tissue from oxidative damage.

The neuron protective potential of flavonoids is well reported and dietary supplementation studies have highlighted their potential to influence cognition. The special physiology structure, the blood brain barrier, and the high lipid content of brain tissue are possibly the reasons for the metabolism of exogenous antioxidants (including antioxidant flavonoids) in brain, which differs from that in other tissues. Interestingly, in brain tissue, the enhancement of antioxidant biomarkers and induction of gene expression were only detected in the high FV juice dosage treatment group. These data indicate that a longer dietary intervention or a higher dosage of FV juice might be essential to achieve enhancing effects of the antioxidant capacity in the brain.

The gene expression of GCLC and NQO1exhibited a slight decreasing trend in rat colon after high dosage of F**V** juice treatment, but there was no statistically significant difference among the groups. Furthermore, expression of the GSTP1, GSTA2, GSTM2, and GCLM genes was not affected by FV juice treatment in colon. The study by Soyalan *et al.* (2011) also reported unchanged or down-regulated GCLC and NQO1 gene expression in apple juice-treated rat colon. Other authors also reported that clear or cloudy apple juice intervention did not significantly change transcript levels of glutathione-associated enzymes in epithelial cells of rat distal colon tissues (Barth*et al.*, 2005, 2007).

Several limitations should be mentioned for the present study. First, we did not identify if changes of antioxidants content were due to antioxidant vitamins, polyphenols or their metabolites, in tissues. Therefore, we cannot explain whether the antioxidant vitamins or polyphenols or their synergistic effects contributed to the elevation of oxidative damage defense in rats. Second, we have no data about the changes of ROS in rat serum or liver, brain and colon tissues. Thus, we cannot discuss the associations of antioxidant content with ROS levels in the serum and organs in detail. Third, the dosage used for intervention was determined according to the daily FV intake recommendation for humans. However, variation between species in food metabolism should ultimately influence the reaction of body antioxidant profile to FV juice intervention, which limits the extrapolation of the data. To assess these issues, further studies are needed.

## Conclusion

FV juice supplementation was effective in enhancing antioxidant capacity due to the elevation of multiple antioxidants in serum and tissues of rats. However, the influence of FV juice on antioxidant gene expression was tissue-dependent. GCLC and NQO1 transcript levels were sensitive to FV juice intervention, especially in liver and brain.

## Supplementary Material

The following online material is available for this study:

Table S1 - Food menu used for dietary intervention.

Table S2 - Content of vitamins and flavonoids in fruits and vegetablesused for juice preparation.

Table S3 - Organ coefficient of rats after fruit and vegetable (FV) juice dietary intervention for 5 weeks.
