# Supplementary material for: Vegetable and fruit juice enhances antioxidant capacity and regulates antioxidant gene expression in rat liver, brain and colon
Source: Genet Mol Biol. 2017 Mar 20;40(1):134–41. doi: 10.1590/1678-4685-GMB-2016-0159 (PMC5409777; doi:10.1590/1678-4685-GMB-2016-0159)
Supplement: Supplementary file 1 [file 1415-4757-gmb-1678-4685-GMB-2016-0159-Suppl01.pdf]

Table S1- Food menu used for dietary intervention.

Day 1	Day 2	Day 3
Carrot juice	Tomato juice	Broccoli juice
Celery juice	Onion juice	Green Pepper juice
Grape juice	Grape juice	Grape juice
