# Supplementary material for: Vegetable and fruit juice enhances antioxidant capacity and regulates antioxidant gene expression in rat liver, brain and colon
Source: Genet Mol Biol. 2017 Mar 20;40(1):134–41. doi: 10.1590/1678-4685-GMB-2016-0159 (PMC5409777; doi:10.1590/1678-4685-GMB-2016-0159)
Supplement: Supplementary file 2 [file 1415-4757-gmb-1678-4685-GMB-2016-0159-Suppl02.pdf]

Table S2- Content of vitamins and flavonoids in fruits and vegetables (FV) used for juice preparation (mg/kg of body weight, mean or mean ± SE)

	Onion(purple)	Broccoli	Carrot	Pepper	Celery	Tomato	Grape (Kyoho)
Vitamin C	80	510	160	720	80	190	40
β-carotene	0.2	72.1	40.1	3.4	3.4	5.5	0.3
Total Vitamin E	1.4	9.1	--	5.9	13.2	5.7	3.4
Quercetin	85.9 ± 14.1	61.7 ± 10.6	37.5 ± 9.1	17.9 ± 1.7	12.4 ± 2.8	20.9 ± 0.2	10.68 ± 1.86
Kaempferol	< 2.0	< 2.0	4.8 ± 1.1	< 2.0	< 2.0	10.1 ± 0.4	< 0.2
Luteolin	<2.0	< 2.0	47.7 ± 0.6	20.9 ± 2.8	11.6 ± 1.1	11.3 ± 6.7	< 0.2
Apigenin	51.9 ± 6.1	12.9 ± 0.5	< 4.0	69.6 ± 14.3	69.7 ± 5.3	< 4.0	< 0.4
Total flavonoids	210.7	74.6	150.3	126.3	93.7	68.0	22.48

Data based on the reports of Wei, *et al.*(2009) Flavonoid in 30 kinds of vegetable sold in Beijing. Chin J Food Hygiene, 21:415-417; Guo, *et al.*(2008) The flavonoid content of common fruits in China. Acta Nutrimenta Sinica, 30:130-135; Yang, *et al.* (2009) China Food Composition. 2th edition.Peking University Medical Press.
