# Supplementary material for: Vegetable and fruit juice enhances antioxidant capacity and regulates antioxidant gene expression in rat liver, brain and colon
Source: Genet Mol Biol. 2017 Mar 20;40(1):134–41. doi: 10.1590/1678-4685-GMB-2016-0159 (PMC5409777; doi:10.1590/1678-4685-GMB-2016-0159)
Supplement: Supplementary file 3 [file 1415-4757-gmb-1678-4685-GMB-2016-0159-Suppl03.pdf]

Table S3 -Organ coefficient of rats after fruit and vegetable (FV) juice dietary intervention for 5 weeks (n=12 for each group).

Group	Brain/weight	Heart/weight	Spleen/weight	Liver/weight	Kidney/weight	Testes/weight
Control	0.400 ± 0.000	0.300 ± 0.000	0.200 ± 0.000	2.600 ± 0.300	0.700 ± 0.100	1.000 ± 0.300
Low FV juice	0.300 ± 0.000	0.300 ± 0.000	0.200 ± 0.000	2.600 ± 0.300	0.700 ± 0.100	0.900 ± 0.100
High FV juice	0.300 ± 0.002	0.300 ± 0.003	0.200 ± 0.004	2.700 ± 0.270	0.700 ± 0.058	0.900 ± 0.180
